# Peer Review of “Viability of Mobile Forms for Population Health Surveys in Low Resource Areas (Preprint)”

**DOI:** 10.2196/64797

**Published:** 2024-09-19

**Authors:** Daniela Saderi, Laetitia Bert, Rakesh

**Affiliations:** 1 PREreview, Portland, OR, United States; 2 Laval University, Quebec City, QC, Canada

**Keywords:** health surveys, health data, mobile forms, Philippines

*This is a peer-review report submitted for the preprint “Viability of Mobile Forms for Population Health Surveys in Low Resource Areas.”*

This review is the result of a virtual collaborative live review discussion organized and hosted by PREreview and JMIR Publications. The discussion was joined by 19 people: 1 author, 2 facilitators, 3 members of the JMIR Publications team, and 13 live review participants. Dr Aishah Ibrahim wished to be recognized for their participation in the live review discussion, even though they have not contributed to authoring the review below. We thank all participants who contributed to the discussion and made it possible for us to provide feedback on this preprint.

## Summary

The study [1] aimed to evaluate the preference for and usability of a custom mobile form software in conducting large-scale population health surveys among volunteer surveyors in low-resource communities in the Philippines. Using convenience sampling, the authors conducted pilot testing and surveys in diverse communities, leading to the development of a user-friendly mobile form software with offline functionality and time tracking. Field testing involved training local surveyors to use the software for health-related surveys, followed by a data collection and analysis phase.

The primary finding indicates that the custom mobile form software is a viable method for large-scale population health surveys in low-resource environments, meeting key needs of offline functionality, user-friendliness, and timing metrics tracking. Initially, 40% of participants from pilot interviews preferred paper due to perceived ease and speed, but after minimal usage, 70% of surveyors found the mobile forms easier and faster to complete.

This research highlights the practical usability of mobile form software in low-resource settings, presenting significant implications for global health initiatives. Its cost-effectiveness compared to traditional paper-based approaches enhances equity in population health studies, facilitating data collection in low-resource settings. The shift in surveyor preferences extends to more complex surveys, making this study a valuable contribution to the field.

This study’s strengths lie in its human-centered approach and comprehension of preferences and usability. However, limitations include a small sample size of surveyors testing the form, lack of replicability, absence of clear limitations and ethical disclosures, and occasional results that do not consistently align with the main topic or question.

Below, we list some concerns that were brought up in the live review, and when possible, we attempt to provide suggestions for addressing them.

## Major Concerns and Feedback

- Rationale of the approach: Reviewers had some questions about the rationale behind the choice of the approach. Was there an initial hypothesis that was tested? If so, can the authors explain the rationale in more detail?
- General clarity: The language used was straightforward, with simple and short sentences, so was generally very easy to follow. However, several reviewers found the manuscript very descriptive and lacking critical analysis/reflection (more on this later in the review). Furthermore, some parts of the article could benefit from restructuring the text (moving text to different sections). For example, it is recommended that the authors consider moving the findings described in the Methodology section to the Results section. Authors may also consider streamlining the manuscript to ensure the same result is not repeated multiple times in the same section, which can be confusing for the reader.
- More methodological details: While the study outlines the general approach used in the pilot interviews and field testing, it would be helpful to add detailed methodological specifics, like the criteria for selecting survey sites and surveyors, the precise training process for surveyors, the number and conditions of interviews, the demographic of the population tested, and the kind of interview method that was used.
- Descriptive results, vague language, unsupported conclusions: The interpretation of the data seems primarily positive toward mobile forms, but it might be somewhat biased due to the lack of objective measures and control groups—the conclusions are largely based on subjective feedback rather than on a comprehensive analysis of performance metrics. This is an important limitation of the study that should be at least recognized. For example, the sentence “The surveyors mostly used their phones for Social Media and Messaging apps. This indicated that these surveyors were reasonably comfortable using their phones.” is a conclusion based on general observation rather than on quantitative assessment. Another example: “Surveyors interviewed were chosen through convenience sampling”; what did the authors mean by this? More information would be needed to better understand how the selection of surveyors was done.
- Similarly, more details are needed about the context of the validity of the research. The ease of training to use the app may not account for varying levels of technological literacy or familiarity among different populations. The conclusion that mobile forms are preferred might be overreaching if generalized beyond the specific demographic and geographic context of the study.
- Furthermore, more empirical support for the conclusion about the potential for broader adoption and preference for mobile forms with increased usage may need more explanation. This statement is based on a hypothesis rather than concrete data from the study. Without objective measures or comparisons to standard benchmarks, conclusions about the ease of use of the mobile app are subjective and may need to be nuanced.
- If data were collected using more robust and established methods for usability testing (eg, task completion time, error rate analysis, validated usability/acceptability questionnaires), the reviewers recommend they be added to the manuscript.
- Finally, the reviewers recommend removing subjective/nonquantitative words to describe the results, such as “good,” “important,” which can guide the reader to misinterpret (and even overinterpret) the results.
- More technical information: The study doesn’t provide in-depth information about the technical aspects of the mobile form software (eg, what language was used to write the code, the code itself). Without this information, replicating the software for a similar study would be challenging. If readers are unable to access the source code used to generate the software, the reproduction and validation of the results would not be possible. The reviewers suggest that the authors consider sharing the source code on GitHub with an open-source license so that others are able to investigate the code, build upon it, and adapt it to their needs so that other groups with the same issues can benefit from this work too.
- Ethics and privacy: Reviewers had several concerns about ethical and privacy issues related to the study. They asked if the mobile app was Health Insurance Portability and Accountability Act compliant and if it had obtained institutional review board approval. Furthermore, the reviewers expressed concern about data privacy for the people who were surveyed through the app. Where were the data stored? Were there ways to secure the data collected on private phones so that they could not be stolen easily?
- Study limitations: Reviewers identified several limitations of the study and suggest that they be discussed in a separate section of the Discussion so that the reader can easily access them. The most important limitations include geographic and demographic limitations, sample selection, lack of a control group, potential technological familiarity and bias (eg, are the people developing the tool the same as the ones conducting the survey?), depth of usability testing, and software development process. Furthermore, although the findings show that there is a dominant interest in mobile forms, the issue of lack of phone ownership, poor internet access, typing speed, and the educational status of the participants should be properly discussed.

## Minor Concerns and Feedback

- Software like REDCap and SurveyMonkey can work offline and can time questions. It would be helpful to compare this newly developed software with existing ones with comparable features.
- Some reviewers wondered if the authors quantified differences in the degree of numerical literacy, language literacy, and technological literacy among the surveyors as factors that could have influenced the speed of filling the mobile forms.
- One of the findings was that a portion of the surveyors were not found to be proficient with modern technology. Some reviewers wondered if the authors saw a correlation between technological proficiency and age. It would be interesting to show if that was the case.
- It would be helpful to know whether informed consent was obtained from the surveyors.
- More information about the research conditions in this context would be helpful (high school internship in the company). Also, sentences like the following one don’t help the reader understand the scientific context or topic: “Since Gawad Kalinga builds free housing in ten thousand locations across the Philippines, it can reach over one million households and mobilize many volunteers.” The reviewers suggest that authors more clearly and specifically state what they want to communicate, in that case presumably that the partner wants to reach respondents on a bigger scale.
- In Figure 6, histogram and summary statistics in text could supplement the visualization.
- The reviewers praise the data visualization, as the authors made it easy for readers to grasp the results. However, higher image resolutions would help improve Figures 1 and 3. Some wondered how Figure 1 supports the argument.
- It would be helpful to have a table summarizing the characteristics of participants.
- In the Introduction, the authors mention there were 33 surveyors, but in the figures, it looks like there were 50.
- Figure 2 should be under the Results section instead of Methods.
- Figure 2 and several subsequent ones: The captions should describe the figures and not interpret the results. Interpretation of the results should be reserved for the Results section (to a certain extent) and for the Discussion.
- If data are comparable, it would be useful to have pre- and postpreference for mobile forms presented in the same figure for comparison, perhaps using different colors for clarity.
- In Figure 4, regarding the location of the study, it would be better either in the Introduction section or primary paragraphs of the Methodology.
- It would be important to include how many surveyors were interviewed right at the beginning of the Methodology section rather than waiting until later in the manuscript.
- Were there any problems regarding the battery life/charging of the mobile phones? How was this dealt with? Were surveyors provided with a charged power bank to overcome a potential lack of power?
- A reviewer suggested the addition of a voice command to the digital survey as a way to collect qualitative research not only for research questions in future research, like open-ended and closed-ended questions.
